# Gradually elevated expression of Gankyrin during human hepatocarcinogenesis and its clinicopathological significance

**DOI:** 10.1038/srep05503

**Published:** 2014-07-07

**Authors:** Hongbiao Jing, Guoming Zhang, Lingsheng Meng, Qingda Meng, Haiying Mo, Yanhong Tai

**Affiliations:** 1 Department of Pathology, the General Hospital, Jinan Military Command, Jinan, China; 2 Department of Cardiology, the General Hospital, Jinan Military Command, Jinan, China; 3 Laboratory Department, the Sixth Hospital of Jinan, Zhangqiu, China; 4 Department of Pathology, Zaozhuang Hospital, Zaozhuang Mining Group, Zaozhuang, China; 5 These authors contributed equally to this work.

## Abstract

Gankyrin is an important oncoprotein that is overexpressed in human hepatocellular carcinoma (HCC). However, the gradual alteration of Gankyrin in successive stages during human HCC development and the mechanism of Gankyrin-mediated hepatocarcinogenesis remain largely unknown. In this study, we evaluated the pattern and level of Gankyrin protein expression using immunohistochemistry in various liver tissues, including normal liver, chronic hepatitis, cirrhosis, adenomatous hyperplasia (AH), and HCC tissues, to analyze its clinicopathological significance. Furthermore, we stably transfected the shRNA-Gan vector, which targets human Gankyrin, into HepG2 cells to assess the role of Gankyrin in cell proliferation and tumorigenicity. The expression level of Gankyrin in the cytoplasm, nucleus, and whole cell was gradually elevated during consecutive stages of hepatocarcinogenesis. The nuclear Gankyrin level in AH was significantly higher than that in normal liver, chronic hepatitis, and cirrhotic tissues. The cytoplasmic, nuclear, and total cellular Gankyrin expression levels in HCC were significantly correlated with capsular invasion and intrahepatic metastasis. Silencing Gankyrin expression using shRNA-Gan repressed tumor cell proliferation, tumorigenicity, migration, and invasion in vitro. Our findings demonstrate that Gankyrin is aberrantly expressed beginning at the initiation stage and plays an important role in the initiation, promotion, and progression of hepatocarcinogenesis.

Hepatocellular carcinoma (HCC) is the fifth most common cancer and third most common cause of cancer-related death worldwide^1^. Therefore, it is critical to elucidate the mechanisms of hepatocarcinogenesis and to identify molecular targets for HCC diagnosis, prevention, and therapy.

Hepatocellular carcinogenesis is a multistep process involving the activation of various oncogenes and the inactivation of tumor suppressor genes through which normal hepatocytes are sequentially transformed into their malignant counterparts^2^,^3^. External factors that contribute to HCC development are infection by hepatitis B or C, chronic alcohol intake, exposure to aflatoxin, and chronic liver disease of any type. These factors can lead to cirrhosis and adenomatous hyperplasia (AH), culminating in HCC with possible metastatic disease^4^. Although this multistep hepatocellular carcinogenesis model has been well established histopathologically and clinically, the molecular mechanisms at each stage (chronically diseased liver, cirrhosis, AH, and HCC) remain elusive.

Gankyrin, also referred to as PSMD10, p28GANK, or p28, has been identified as a novel oncoprotein that is composed of 7 ankyrin repeats and is highly conserved in mammals^5^,^6^,^7^. It has been documented that these ankyrin repeats are the functional domains that are involved in protein–protein interactions. By binding to retinoblastoma (Rb) protein and cyclin-dependent kinase 4 (CDK4), Gankyrin facilitates the phosphorylation and degradation of Rb and activates the E2F family of transcription factors, thus promoting tumorigenicity and cancer cell proliferation^5^,^8^,^9^. Furthermore, by interaction with the ubiquitin protein ligase, murine double minute 2 (Mdm2), Gankyrin promotes p53 ubiquitylation and subsequent proteasomal degradation, leading to the inactivation of p53-dependent apoptotic genes and stimulating the tumorigenic transformation of cells^10^,^11^,^12^.

To evaluate the role of Gankyrin in HCC development and analyze its clinicopathological significance, we examined the level and pattern of Gankyrin protein expression in various liver tissues at each stage of the multistep process of human hepatocarcinogenesis using immunohistochemistry (IHC). Furthermore, to evaluate the effects of Gankyrin downregulation on tumor biological behavior, we used a plasmid vector to express a short-hairpin RNA (shRNA) targeted against Gankyrin mRNA in HepG2 human HCC cells (ATCC, Rockville, MD).

## Results

### Clinicopathological profiles of the patients

The study group comprised 312 patients with HCC (282 males and 30 females), 33 patients with benign hepatic hemangioma, and 58 patients with AH. The clinicopathological characteristics of the patients with HCC are summarized in Table 1. The ages of the HCC patients at the time of surgery ranged from 19 to 79 years (mean, 51.27 ± 10.83 years). Of the 312 patients with HCC, 193 had follow-up data available, with follow-up intervals ranging from 2 to 101 months. Histological findings compatible with normal liver, chronic hepatitis without cirrhosis, and cirrhosis with or without chronic hepatitis were observed in the non-cancerous liver tissues from 6, 52, and 254 HCC patients, respectively. In addition, AH was observed in adjacent liver samples from 49 HCC patients. Consequently, 107 AH tissue samples, 49 of which were from adjacent tissues of HCC patients and 58 of which were from the fine needle aspiration tissue samples, were collected in present study.

### The expression level of Gankyrin is gradually elevated throughout the consecutive stages of hepatocarcinogenesis

**Figure 1:**
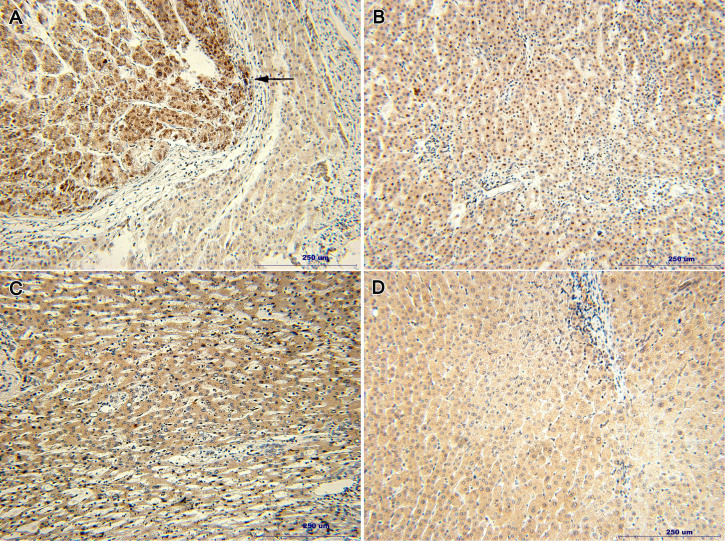
Gradual changes in the level and pattern of Gankyrin expression during the successive stages of hepatocarcinogenesis, as determined using IHC. (A) Gankyrin staining was strong in both the cytoplasm and nucleus of HCC cells (arrow). (B) Gankyrin was moderately expressed in both the cytoplasm and nucleus of AH cells. (C and D) In contrast, Gankyrin was weakly expressed in the cytoplasm of hepatocytes and occasionally expressed in the nucleus of hepatocytes in chronic hepatitis (C) and cirrhosis (D) tissues.

**Figure 2:**
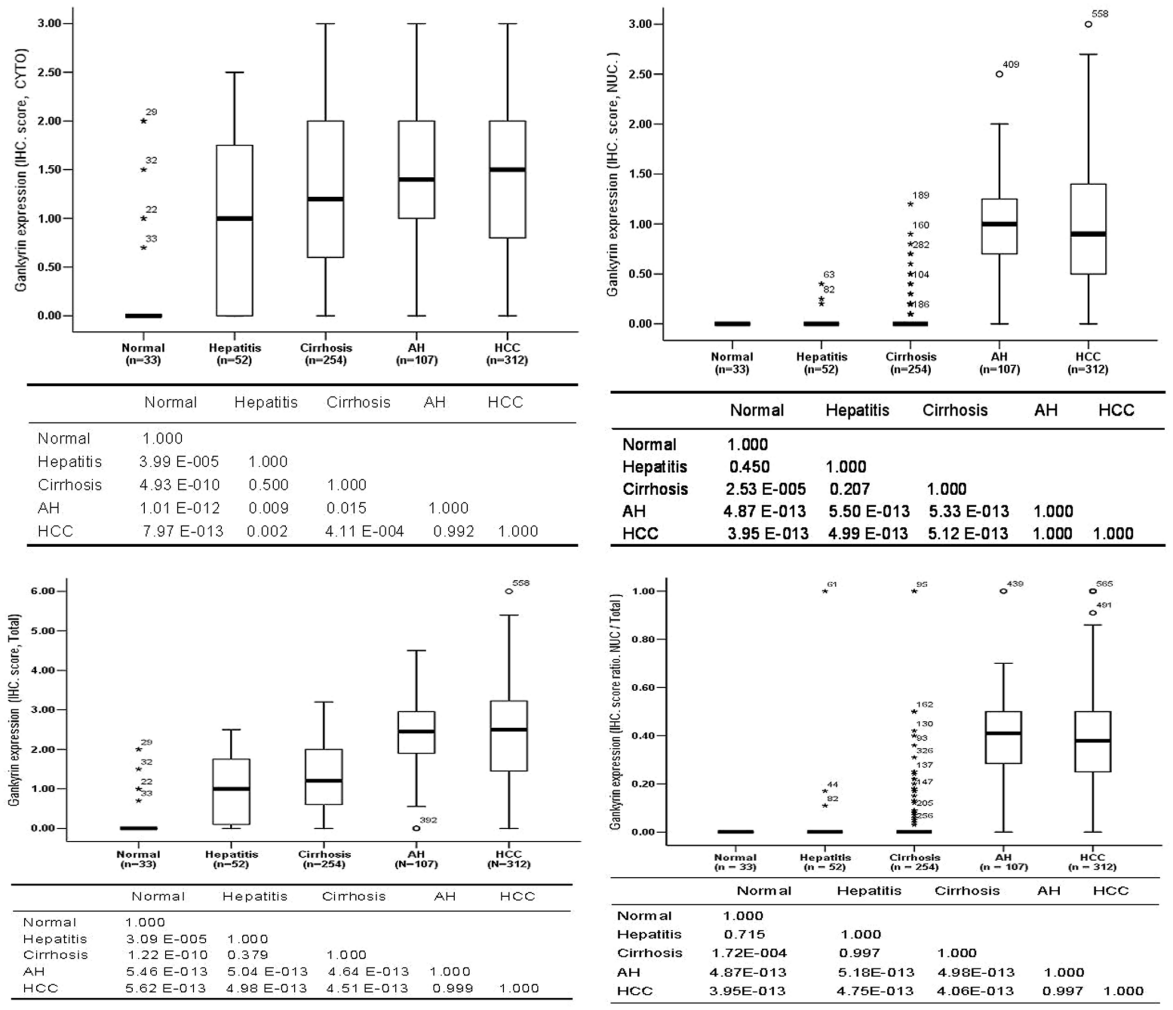
Boxplot of the immunostaining scores for Gankyrin in the cytoplasm, nucleus, whole cell, and nuclear expression vs. total expression ratio in the normal liver, hepatitis, cirrhosis, AH, and HCC specimens utilized in this study. A statistical analysis of Gankyrin expression was performed, with the P-value for the difference among the experimental groups shown (bottom parts).

To study the temporal alteration of Gankyrin expression during hepatocarcinogenesis, liver samples at successive stages of HCC development, including normal liver, chronic hepatitis, cirrhosis, AH, and HCC tissues, were immunohistochemically stained using an anti-Gankyrin antibody. Both the expression level and topological location of Gankyrin in the cells were systematically evaluated at the subcellular level. In HCC and AH samples, Gankyrin was diffusely localized throughout both the cytoplasm and nuclei of tumor cells with various staining intensities, from weak to severe positivity (Figure 1A and B). In chronic hepatitis and cirrhosis tissues, Gankyrin was weakly present in the cytoplasm of hepatocytes, whereas nuclear immunostaining was also occasionally observed (Figure 1C and D). Very weak cytoplasmic positivity for Gankyrin was observed in 6 of the 33 normal liver tissues from the resected benign hepatic hemangioma samples; the remaining 27 normal liver tissues were Gankyrin-negative in both the cytoplasm and nucleus. Gankyrin expression was absent in bile duct cells, blood endothelial cells, and other interstitial cells in all the above-mentioned liver tissues. As shown in Figure 2, Gankyrin expression progressively increased as follows: normal liver < chronic hepatitis < cirrhosis < AH and HCC tissues (corresponding to the sequential stages of hepatocarcinogenesis); this pattern was observed in cytoplasmic, nuclear, and total cellular expression (P = 4.78 E-016, P = 9.70 E-096, and P = 9.45 E-052, respectively). Beginning at the chronic hepatitis stage, the levels of both cytoplasmic Gankyrin expression and total cellular Gankyrin expression were significantly higher than in normal liver tissues, suggesting that Gankyrin overexpression begins at the initiation stage, which is the earliest stage of hepatocellular carcinogenesis. No significant difference was observed between the Gankyrin expression in AH and HCC tissues, regardless of whether expression was scored at the cytoplasmic, nuclear, or total cellular level. Intriguingly, nuclear Gankyrin staining was absent in normal liver tissues, occasionally observed in chronic hepatitis and cirrhosis tissues, and mainly detected in AH and HCC tissues, where its levels were significantly increased compared with normal liver, chronic hepatitis, and cirrhosis tissues. Moreover, the increasing tendency of nuclear Gankyrin expression vs. the total expression ratio was similar to that of nuclear staining in the sequential stages of hepatocarcinogenesis (Fig. 2). These observations indicate that Gankyrin nuclear localization is not just the result of increased expression but is also due to the active localization of a localized tumor-related function, which may contribute to the malignant transformation of hepatocytes during carcinogenesis.

### Upregulated Gankyrin expression is correlated with capsular invasion and intrahepatic metastasis in HCC

**Figure 3:**
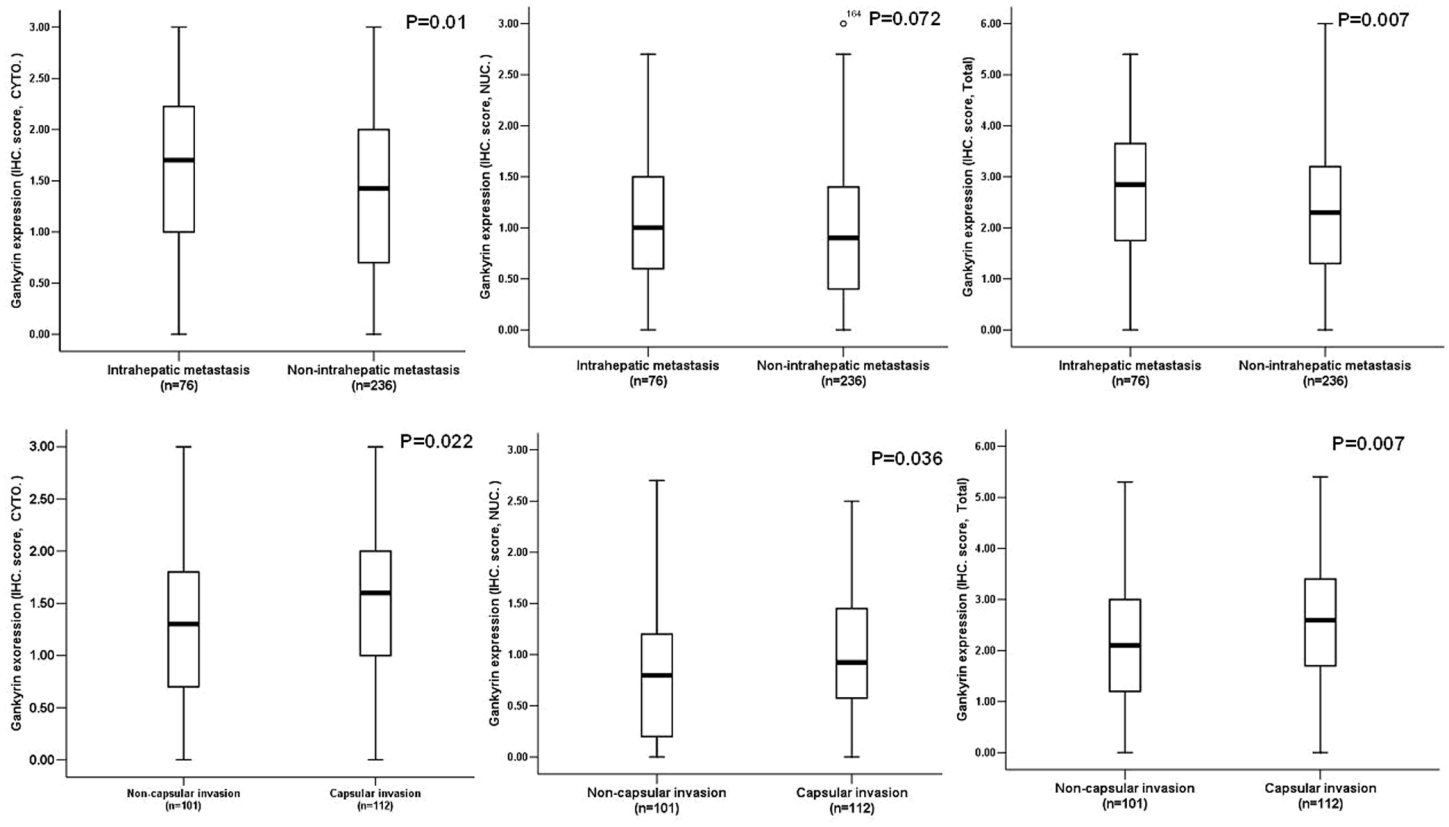
The intratumoral overexpression of Gankyrin scored at the cytoplasmic, nuclear, and total cellular levels was statistically correlated with capsular invasion and intrahepatic metastasis in HCC patients.

We compared Gankyrin expression scored at the cytoplasmic, nuclear, and total cellular levels with various clinicopathological parameters in patients with HCC. The cytoplasmic, nuclear, and total cellular Gankyrin expression levels were all significantly correlated with capsular invasion, and both the cytoplasmic and total cellular expression levels were correlated with intrahepatic metastasis (Figure 3), suggesting that Gankyrin overexpression is an important event during the progression of HCC. Moreover, nuclear expression of Gankyrin was significantly associated with clinical stage (P = 0.010). However, there was no relationship between Gankyrin expression at the cytoplasmic, nuclear, or total cellular level and other clinicopathological parameters, including tumor size, differentiation status, number of tumors, intravascular tumor thrombus, capsule formation, or lymph node metastases.

**Figure 4:**
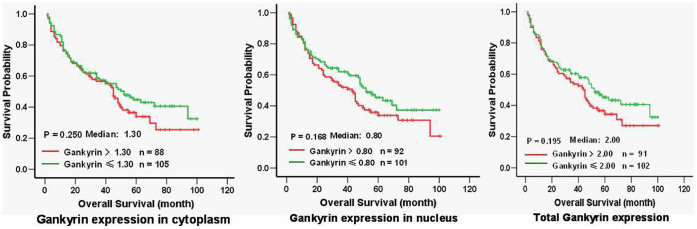
Comparison of the overall survival rates of 193 patients with available follow-up data between the low-Gankyrin and high-Gankyrin groups.

Based on Gankyrin immunostaining, all 193 HCC patients with available follow-up data were divided into a high-expression group and a low-expression group. The HCC patients with Gankyrin expression H-scores that were higher than the median were included in the high-expression group; the remaining patients were included in the low-expression group. As shown in Figure 4, 88, 92, and 91 patients were included in the cytoplasmic, nuclear, and total cellular high-expression groups, respectively. The overall survival of patients in the high-expression group was compared with that of patients in the low-expression group; however, no significant differences were observed, regardless of whether cytoplasmic, nuclear, or total cellular staining was observed.

To reduce the interference due to age-related factors in the cumulative survival analysis, the patients with follow-up data were divided into X, Y, and Z groups. Patients who were within 40 years of age were enrolled into X group, patients whose ages were >40 and ≤60 years were included in Y group, and the remaining patients were included in Z group. In each group, the patients were redivided into a high-expression group and a low-expression group according to Gankyrin expression. The survival assay was performed again, and no significant differences were observed, regardless of whether cytoplasmic, nuclear, or total cellular staining was observed (data not shown).

Considering that Gankyrin exerts its oncogenic function by regulating the phosphorylation of Rb and the ubiquitylation of p53^5^,^8^,^9^,^10^,^11^,^12^, we examined by IHC the expression of p53 and Rb as well as Gankyrin in 193 HCC samples with available follow-up data. Comparing Gankyrin expression at the cytoplasmic, nuclear, and total cellular levels with p53- and Rb-positive nuclear staining levels, we observed no significant correlation of Gankyrin expression with Rb or p53 expression (data not shown).

### Suppression of Gankyrin expression attenuates HepG2 cell proliferation, tumorigenicity, migration, and invasion in vitro

**Figure 5:**
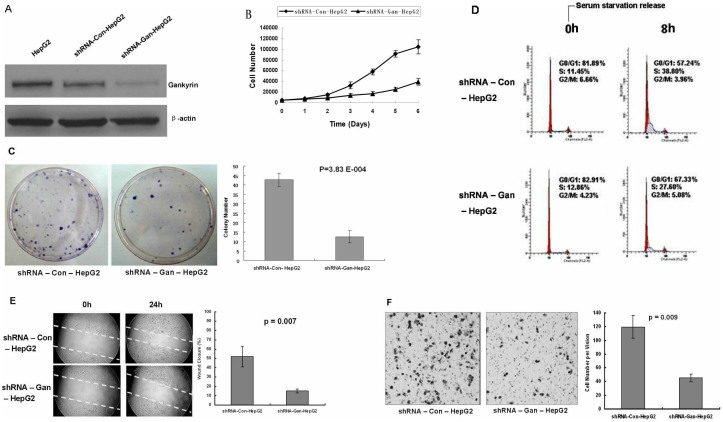
Inhibition of the malignant phenotypes of HepG2 cells by silencing Gankyrin expression in vitro. The results are presented as the mean of three independent experiments, with the corresponding standard deviation (B, C, D, E, and F). (A) Western blot analysis of Gankyrin protein expression in shRNA-Con-HepG2 and shRNA-Gan-HepG2 cells. β-actin was used as an internal control. Gankyrin protein expression was significantly lower in shRNA-Gan-HepG2 cells than in shRNA-Con-HepG2 cells. (B) Growth curves for shRNA-Con-HepG2 and shRNA-Gan-HepG2 cells. shRNA-Gan-HepG2 cells were significantly fewer than shRNA-Con-HepG2 cells (second day: 15533.3 ± 1137.25 vs. 9500.0 ± 1212.44, P = 0.003; third day: 32733.3 ± 6404.95 vs. 13933.3 ± 2936.55, P = 0.010; forth day: 58033.3 ± 5204.17 vs. 17466.7 ± 3350.12, P = 3.43 E-04; fifth day: 91500.0 ± 6378.87 vs. 25100.0 ± 4190.47, P = 1.13 E-04; sixth day: 104833.3 ± 13089.05 vs. 39300.0 ± 5692.98, P = 0.001). (C) The tumorigenicity of shRNA-Gan-HepG2 cells was significantly lower than that of controls, as evaluated using the soft agar colony formation assay. (D) A cell cycle assay performed by flow cytometry. Downregulation of Gankyrin expression in HepG2 cells led to the prohibition of cell cycle progression from G1 to S phase. (E) Representative images of wound healing assays showing that the migratory capability of shRNA-Gan-HepG2 cells was significantly suppressed compared to controls. (F) The invasion potential was analyzed by a Matrigel invasion assay. The invasion of shRNA-Gan-HepG2 cells was statistically decreased compared to controls.

To investigate the possible effect of Gankyrin-shRNA on Gankyrin expression in vitro, we established stable clones by transfecting vector-based shRNAs targeted against Gankyrin in HepG2 cells, which endogenously express Gankyrin. We then analyzed the expression level of Gankyrin in shRNA-Gan-HepG2 cells and shRNA-Con-HepG2 cells using Western blotting. As shown in Figure 5A, the level of Gankyrin protein in shRNA-Gan-HepG2 cells was obviously reduced compared with that in shRNA-Con-HepG2 cells, suggesting that shRNA-Gan could inhibit Gankyrin expression in HepG2 cells in vitro. Consequently, shRNA-Gan-HepG2 cells were utilized in further analyses.

To evaluate the effects of Gankyrin downregulation on HepG2 cell biology in vitro, we performed cell growth, soft agar colony formation, cell cycle, migration, and invasion assays. As shown in Figure 5B, shRNA-Gan-HepG2 cells grew much more slowly than the controls, and a statistical analysis exhibited a significant difference beginning on the second day, indicating that the downregulation of Gankyrin expression can inhibit the proliferation of HepG2 cells in vitro. The soft agar colony formation assay revealed that shRNA-Gan-HepG2 cells, shRNA-Con-HepG2 cells yielded 12.3 ± 3.21 and 42.7 ± 3.51 colonies, respectively, after 2 weeks (P = 3.83 E-004; Figure 5C), demonstrating that downregulation of Gankyrin expression can suppress the tumorigenicity of HepG2 cells in vitro. The flow cytometry analysis demonstrated that cell populations in the G0/G1 phase were significantly larger for the shRNA-Gan-HepG2 cells than the shRNA-Con-HepG2 cells (67.33% ± 6.74 vs. 57.24% ± 3.90, p = 0.027; Fig. 5D). Hence, the shRNA-mediated downregulation of Gankyrin expression in HepG2 cells leads to the prohibition of cell cycle progression from G1 to S phase. In addition, wound healing and Matrigel assays were used to investigate the effect of Gankyrin downregulation on cell motility and invasion in vitro. The results showed that the migration and invasion potential of Gankyrin knocked down cells was significantly reduced in comparison with the controls (p = 0.007, p = 0.009, respectively; Fig. 5E and F). Taken together, these data reveal that downregulation of Gankyrin expression can inhibit the malignant phenotype of HepG2 cells.

## Discussion

HCC develops in a well-defined multistep process, from normal liver to chronic hepatitis, cirrhosis, AH, and finally to HCC^2^,^3^. However, the molecular mechanisms of multistep hepatocarcinogenesis remain unclear. Previous studies have shown that Gankyrin is a critical oncoprotein that is frequently overexpressed in human HCC and that is involved in the progression of HCC via the degradation of Rb and elimination of p53^5^,^13^,^14^,^15^,^16^,^17^,^18^,^19^,^20^. Few reports have described the early initiating events in hepatocarcinogenesis. In the well-established rat model of HCC induced by diethylnitrosamine (DEN) and other chemicals, it was shown that Gankyrin overexpression, pRb degradation, and the hypermethylation of CDKN2A and TP53 are the initiating events that promote the multistep process of hepatocarcinogenesis^14^,^16^,^19^. However, the chronic hepatitis, cirrhosis, AH, and HCC tissues investigated in the above-mentioned studies were obtained from rats rather than humans. Thus, the present study is the first to examine the pattern and level of Gankyrin expression in a series of human liver tissues, including normal liver, chronic hepatitis, cirrhosis, AH, and HCC tissues, using immunohistochemistry. We found that the level of Gankyrin protein expression in the cytoplasm, nucleus, and whole cell was progressively and significantly increased during the course of human hepatocarcinogenesis. Consequently, we have confirmed for the first time that aberrant Gankyrin expression plays an important role in the early stages of human hepatocarcinogenesis, as previously determined in a rat HCC model and a mouse cirrhosis model^14^,^16^,^19^. In addition, we found that Gankyrin overexpression began in the chronic hepatitis stage, corresponding to the initiation stage of human hepatocarcinogenesis.

Studies on the expression pattern of Gankyrin with regard to its subcellular localization and its clinicopathological significance were reported by Tan et al. based on 55 cases of HCC^13^. These studies showed that Gankyrin is overexpressed in the cytoplasm as well as the nucleus of HCC cells, and the probability of nuclear Gankyrin expression in HCC was statistically higher than that in para-carcinoma hepatic tissues. However, to the best of our knowledge, no studies to date have reported the successive alteration of Gankyrin expression patterns and roles during the course of hepatocarcinogenesis. Consequently, we evaluated the immunostaining patterns of Gankyrin during human hepatocarcinogenesis for the first time. We determined that cytoplasmic Gankyrin translocates to the nucleus in AH and HCC tissues, suggesting that the nuclear translocation of Gankyrin is important for transforming non-umorous hepatocytes into neoplastic counterparts during hepatocarcinogenesis.

The differentiation between cirrhosis and AH, which usually occurs in cirrhotic livers, would be clinically valuable for the further treatment of patients because the former is benign, whereas the latter is a precancerous disorder that is known to be more closely associated with an increased risk of malignant transformation and should be treated as a potentially malignant lesion^21^. However, even for the experienced pathologist, determining a reliable diagnosis may be a challenge in some cases, particularly if only small biopsies are available as the main source of tissue for histological assays. At present, the pathological differentiation between these two lesions is mainly based on their morphological features; no specific and sensitive immunohistochemical markers have been found that can be used to differentiate cirrhosis from AH. In the current study, the nuclear immunolabeling of Gankyrin was weak and was present in only a small number of hepatocytes in cirrhotic tissues, whereas both the incidence and intensity of nuclear Gankyrin expression were markedly greater in AH tissues than in cirrhotic tissues. This result indicates that the nuclear staining of Gankyrin in hepatocytes could be a potentially useful marker for distinguishing cirrhosis from AH; however, this possibility must be confirmed by future studies.

The underlying mechanism that controls the nuclear translocation of Gankyrin in AH and HCC tissues remains elusive. Lu et al. first reported that nuclear Gankyrin was significantly more frequently observed in HCC than in matched para-carcinoma tissues. These authors hypothesized that Gankyrin promotes cellular proliferation by targeting Rb protein. Because hypophosphorylated Rb is typically localized in the nucleus of hepatocytes, the hyperphosphorylation of Rb may require the translocation of Gankyrin into the nucleus to perform its adapter function by targeting Cdk4 to Rb^13^.

Aberrant expression of oncogenes leads to the initiation, promotion, and progression of a variety of cancers, and an understanding of the mechanisms underlying the changes in oncogene expression that occur during cancer onset and progression is of particular interest for the development of cancer diagnostic, preventive, and therapeutic strategies^22^. In addition to AH patients, most chronic hepatitis and cirrhosis patients are at high risk for liver cancer, which indicates that the prevention of HCC at the chronic hepatitis and cirrhosis stages is rather important^4^. Considering that Gankyrin expression levels progressively increase within multistep HCC carcinogenesis and that Gankyrin overexpression begins at the chronic hepatitis stage, we hypothesize that silencing Gankyrin at the chronic hepatitis or cirrhosis stage might impair hepatocarcinogenesis; this possibility will be further explored in future studies.

An analysis of the association between Gankyrin expression and clinicopathological characteristics in 312 HCC patients demonstrated that Gankyrin expression was significantly correlated with intrahepatic metastasis and capsule infiltration, both of which are markers for poor HCC prognosis^23^. These results indicated that Gankyrin plays an important role in the progression of HCC. Subsequent biological phenotypic experiments have demonstrated that shRNA-mediated downregulation of Gankyrin significantly decreased the malignancy of HepG2 cells in vitro by inhibiting cell proliferation, tumorigenicity, migration, and invasion. Therefore, Gankyrin can be considered a useful target for HCC gene therapy.

It was previously reported Gankyrin controls the activities of Rb and p53 in cultured U-2 OS cells and that the expression of Gankyrin is correlated with Rb in HCC tissues^5^,^10^,^13^. However, the immunostaining of Gankyrin was not statistically correlated with that of p53 or Rb in clinical HCC samples in present study, which is consistent with the previous reports of Umemura et al. and Fu et al.^17^,^18^. Several possible reasons for these contradictory findings are presented as follows. First, the inconsistent results may be due to differences in the techniques used or differences in the tumor samples examined, and/or both. Second, as initially suggested, the immunohistochemical p53 protein positivity may not be a marker of p53 gene mutations^24^. Third, given the complex Gankyrin-MDM2-p53 and Gankyrin-CDK4-Rb loops, it is possible that there are other unknown regulators involved in those pathways. Nonetheless, our results demonstrate that it is likely that Gankyrin-induced HCC progression is independent of p53 and Rb status; the relevance of the effects of Gankyrin on p53 and Rb suggested in cultured cells to human HCC progression remains to be fully studied in the future.

In conclusion, our data indicate that Gankyrin not only participates in the initiation and promotion of hepatocarcinogenesis but also participates in its progression. Moreover, Gankyrin is a potential target for the development of novel diagnostic, therapeutic, and preventive strategies for HCC.

## Methods

### Patients and samples

The pathological records of all patients between January 2000 and December 2012 at the Department of Pathology, General Hospital, Jinan Military Command with liver lesions, including chronic hepatitis, cirrhosis, AH, HCC, and benign hemangioma, were reviewed retrospectively. These liver lesions were screened and confirmed using hematoxylin and eosin (H&E) staining. Patients for whom complete clinical information was available and who gave their informed consent to use specimens for research purposes were included in this study. In addition, the included HCC patients had not received chemotherapy, radiation therapy, or any other anticancer treatment before curative hepatectomy, and their surgically resected tumor samples included sufficient para-carcinoma tissues further than 2 cm away from the focus. Moreover, neither carcinoma nor AH tissues were observed at the edge of the para-carcinoma tissues. The patients with benign hepatic hemangioma exhibited normal liver function, and none had a previous history of any liver disease, ongoing liver dysfunction, or any marker for hepatitis B or C. Moreover, the samples of benign hepatic hemangioma had adjacent liver tissues. In total, 312 cases of HCC and 33 cases of benign hemangioma met the inclusion criteria; consequently, these patients were enrolled in the current study. In addition, fine needle aspiration tissues from 58 cases of AH were also included.

The specimens were fixed in 10% neutral buffered formalin, processed routinely, and embedded in paraffin. Sections (4-μm) were stained with H&E for histological evaluation. The diagnosis and histological grade and tumor-node-metastasis (TNM) classification of HCC were based on the general rules for HCC in the fourth edition of the WHO classification of tumors of the digestive system^25^.

Clinicopathological and follow-up data for the patients were extracted from their medical records. Written informed consent was obtained from the patients, and the present study was approved by the Human Ethics Committee of the General Hospital, Jinan Military Command.

### Immunohistochemical staining and evaluation

IHC was performed using the Dako Envision system (Dako, Carpinteria, CA), as described previously^26^. Primary antibodies against human p53 (DO-1, diluted 1:100), Rb (IF 8, diluted 1:100), p-Rb (Ser 807, diluted 1:100), and Gankyrin (H-231, diluted 1:400) were purchased from Santa Cruz Biotechnology, INC., Santa Cruz, CA. Appropriate positive and negative controls were used.

Nuclear or cytoplasmic staining was considered specific Gankyrin immunoreactivity. For p53, Rb, and p-Rb, only nuclear labeling was regarded as positivity. The status of immunostaining was evaluated in a semiquantitative manner by considering both the intensity and percentage of positive cells to generate a histochemical score (H-score), as described previously^26^. For Gankyrin staining, each case was assigned two H-scores, one each for nuclear and cytoplasmic staining. A final total cellular H-score for Gankyrin staining was determined as follows: total cellular score = H-score of nuclear staining + H-score of cytoplasmic staining. A minimum of 100 cells were evaluated to calculate the H-score. All sections were independently evaluated by two experienced pathologists who were blinded to the clinical and pathological data. When the opinions of the two pathologists differed, an agreement was reached by careful discussion.

### Construction of shRNA-Gan and shRNA-Con

The target shRNA sequence for human Gankyrin was 5′-GAG ATC GCT GTC ATG TTA C-3′; the scrambled sequence 5′-CCA GAA GAG CAA TCT GTA C-3′, which does not target any known gene, was used as a negative control. shRNA oligonucleotides were synthesized, annealed, and inserted into the pSR-GFP/Neo vector, which contains an H1 promoter and a green fluorescent protein reporter gene, according to the manufacturer's protocol. All sequences were confirmed by sequencing. The shRNA targeting human Gankyrin and the control shRNA were designated shRNA-Gan and shRNA-Con, respectively.

### Cell culture and transfection

HepG2 cells were maintained in Dulbecco's modified Eagle's medium (DMEM) (Invitrogen) supplemented with 10% FBS in an incubator at 37°C with 90% relative humidity and 5% CO_2_. Synthetic shRNAs (shRNA-Gan and shRNA-Con) were transfected into HepG2 cells using Lipofectamine 2000 (Invitrogen) according to the manufacturer's protocol. Briefly, HepG2 cells were grown to 70–90% confluence in a 6-well plate and then transfected with shRNA-Gan or shRNA-Con at a final concentration of 5 μg/ml. Twenty-four hours later, the medium was replaced with fresh medium containing FBS, and the culture was continued for an additional 48 hours. Finally, the cells were harvested for subsequent analyses. Stably transfected cells were selected using geneticin at a final concentration of 200 μg/ml. The cells that were stably transfected with shRNA-Gan or shRNA-Con are referred to as shRNA-Gan-HepG2 and shRNA-Con-HepG2, respectively.

### Protein extraction and Western blot analysis

Total proteins were extracted from 1 × 10^7^ cells that were grown to 70–90% confluence in cell lysis buffer. After centrifugation at 18,000 × g for 15 minutes at 4°C, the supernatant was collected, and protein was quantified using a Qubit Protein Assay Kit (Invitrogen) following the manufacturer's protocol.

Immunoblotting was carried out three times independently to evaluate the Gankyrin protein expression level. Briefly, 50 μg of protein was separated using 10% sodium dodecyl sulfate-polyacrylamide gel electrophoresis and electrophoretically transferred onto nitrocellulose membranes (Millipore, Bedford, MA). The membranes were blocked with TBST (20 mM Tris, 150 mM NaCl, and 1% Tween, pH 7.6) containing 5% skim milk powder for 2 hours at room temperature. The membranes were then incubated with an anti-human Gankyrin rabbit polyclonal antibody (Santa Cruz Biotechnology, diluted 1:500) at 4°C overnight, followed by incubation with a horseradish peroxidase-conjugated goat anti-rabbit immunoglobulin antibody (Dako) at room temperature for 90 minutes. The reaction products were visualized by enhanced chemiluminescence (Pierce, Rockford, IL). β-actin (Abcam, Cambridge, MA) served as an internal control. Specific protein bands were photographed.

### Cell growth assay

Cells in log phase (shRNA-Gan-HepG2 and shRNA-Con-HepG2) were trypsinized, and single-cell suspensions were created. The cells were then reseeded in 12-well plates at a density of 5 × 10^3^ cells/well in triplicate at Day 0. After 1, 2, 3, 4, 5, and 6 days of incubation at 37°C, the cells in each well were counted using a Coulter Counter (Beckman Coulter, Fullerton, CA). Counts obtained from three wells were averaged. Each study was repeated three times.

### Colony formation in soft agar

A soft agar colony formation assay was performed to assess anchorage-independent growth, which is a feature of in vitro tumorigenicity. Briefly, single-cell suspensions were prepared from successfully transfected cells (shRNA-Gan-HepG2 and shRNA-Con-HepG2) in 0.3% agar containing DMEM and 10% FBS. Then, the mixtures were layered on top of hardened 0.6% agar (prepared with the same medium) in 6-well plates at a density of 1 × 10^4^ cells per well and cultured at 37°C in the presence of 5% CO_2_ for 2 weeks. The cell colonies were stained with 0.05% crystal violet (Sigma, St Louis, MO) and examined using a light microscope. Colonies with diameters larger than 100 μm were counted. The assay was performed with triplicate samples and was repeated three times.

### Cell cycle analysis

Cells were synchronized by serum starvation for 24 hours and stimulated by replacing with complete medium. Then, a flow cytrometric assay was performed to evaluate the cell cycle phase distribution, as previously described^27^. The evaluation of DNA content was performed using a FACScan flow cytometer (Becton Dickinson, Mountain View, CA). Multicycle Software (version 3.1.1, Phoenix Flow System, San Diego, CA) was used for the cell cycle analyses. Each experiment was repeated 3 times.

### Wound healing assay

Cells (shRNA-Gan-HepG2 and shRNA-Con-HepG2) were cultured to a confluent monolayer in 6-well plates. The cell monolayer was cross scratched with a sterile 200-μl pipette tip to form a wound, and the debris was removed by washing the cells with serum-free DMEM. Photographs of the wound area were obtained at 0 and 24 hours after scratching and analyzed using the IPP 6.0 system (Intel, Santa Clara, CA) at a magnification of ×100. The experiments were performed in triplicate.

### Invasion assay

A Matrigel invasion assay was conducted in triplicate for tumor cell invasion analysis, as described previously with minor modification^28^. After incubation for 36 hours, penetrating cells were counted in 10 randomly selected microscopic fields (×200) after being fixed with formalin and stained with crystal violet (Sigma).

### Statistical analysis

The means of continuous variables, such as the Gankyrin staining level between different stages during the multistep hepatocarcinogenesis process, were compared by a one-way analysis of variance (multiple comparisons). The clinicopathological characteristics of HCC and the Gankyrin expression level were compared using the Mann-Whitney U test. Multiple comparisons of cell growth, soft agar, cell cycle, migration, and invasion assays were analyzed using the t test. Cumulative survival curves were calculated using the Kaplan–Meier method. The SPSS 13.0 software package (SPSS Inc., Chicago, IL) was used for the statistical analyses. All statistical tests were two-sided, and P values < 0.05 were considered to indicate statistical significance.

## Author Contributions

H.J., G.Z. and Y.T. designed the experiments; H.J., G.Z., L.M., Q.M., H.M. and Y.T. performed the experiments; H.M. and Y.T. analyzed and interpreted the data; H.J., G.Z. and Y.T. drafted the manuscript. All authors read, commented on, and approved the manuscript.

## Figures and Tables

**Table 1:** Clinicopathological characteristics of patients with HCC

Variables	Cases	Percentage (%)
Age (years)		
≤40	40	12.82
>40, ≤60	207	66.35
>60	65	20.83
Gender		
Male	282	90.38
Female	30	9.62
Tumor size		
≤2 cm	33	10.58
>2 cm	279	89.42
Status of differentiation		
Well differentiated	38	12.18
Moderately differentiated	224	71.79
Poorly differentiated	50	16.03
Clinical stage		
I + II	162	51.92
III	118	37.82
IV	32	10.26
Number of tumors		
Single	269	86.21
Multiple in one lobe	29	9.29
Multiple in more than one lobe	14	4.50
Intrahepatic metastasis		
Yes	76	24.36
No	236	75.64
Lymph node metastasis		
Yes	4	1.28
No	308	98.72
Capsule formation		
Yes	213	68.27
No	99	31.73
Capsular invasion		
Yes	112	35.90
No	101	32.37
Intravascular tumor		
thrombus		
Yes	140	44.87
No	172	55.13
Background liver disease		
“Normal” liver	6	1.92
Chronic hepatitis	52	16.67
Cirrhosis	254	81.41
